# The Congenital Cataract-Linked A2V Mutation Impairs Tetramer Formation and Promotes Aggregation of βB2-Crystallin

**DOI:** 10.1371/journal.pone.0051200

**Published:** 2012-12-06

**Authors:** Jia Xu, Sha Wang, Wei-Jie Zhao, Yi-Bo Xi, Yong-Bin Yan, Ke Yao

**Affiliations:** 1 Eye Center of the 2nd Affiliated Hospital, Medical College of Zhejiang University, Hangzhou, China; 2 State Key Laboratory of Biomembrane and Membrane Biotechnology, School of Life Sciences, Tsinghua University, Beijing, China; 3 Institute of Biophysics, Lanzhou University, Lanzhou, China; Aligarh Muslim University, India

## Abstract

β/γ-Crystallins, the major structural proteins in human lens, are highly conserved in their tertiary structures but distinct in the quaternary structures. The N- and C-terminal extensions have been proposed to play a crucial role in mediating the size of β-crystallin assembly. In this research, we investigated the molecular mechanism underlying the congenital hereditary cataract caused by the recently characterized A2V mutation in βB2-crystallin. Spectroscopic experiments indicated that the mutation did not affect the secondary and tertiary structures of βB2-crystallin. The mutation did not affect the formation of βB2/βA3-crystallin heteromer as well as the stability and folding of the heteromer, suggesting that the mutation might not interfere with the protein interacting network in the lens. However, the tetramerization of βB2-crystallin at high protein concentrations was retarded by the A2V mutation. The mutation slightly decreased the thermal stability and promoted the thermal aggregation of βB2-crystallin. Although it did not influence the stability of βB2-crystallin against denaturation induced by chemical denaturants and UV irradiation, the A2V mutant was more prone to be trapped in the off-pathway aggregation process during kinetic refolding. Our results suggested that the A2V mutation might lead to injury of lens optical properties by decreasing βB2-crystallin stability against heat treatment and by impairing βB2-crystallin assembly into high-order homo-oligomers.

## Introduction

To correctly project the image onto the retina, the human lens is required to be transparent to visible light and avoid scattering, to absorb the harmful UV light and quench the emission pathway and to adjust the refractive index gradient accommodated to the scene with various distances [1]. To fulfill these functional requirements, the lens fiber cells are highly differentiated with the degradation of intracellular organelles and the expression of high concentrations of lens-specific proteins. Particularly, crystallins are the major protein components in vertebrate lens and account for about 90% of the total lens proteins [2]. All members in the crystallin family have similar molecular weight of the subunits, and can be classified as α-, β- and γ-crystallins according to their oligomeric distributions. Both members (αA and αB) of α-crystallin are large oligomers with high polydispersity ranging from 10-mers to 40-mers [3]. β-Cystallins contain four acidic proteins (βA1, βA2, βA3 and βA4) and three basic proteins (βB1, βB2, βB3) with the ability to form homomers or heteromers, while γ-crystallin are exclusively monomeric. In the lens, α-crystallin functions as a molecular chaperone to protect other proteins from aggregation, while β- and γ-crystallins are thought to be the lens structural proteins to maintain the optical properties of the lens [2], [4], [5]. Due to the lack of protein turnover in lens fiber cells, crystallins have to maintain soluble and stable throughout the whole lifespan of the organism. Any alternations in solubility and/or stability of crystallins can lead to cataract, which is resulted from the interference of visible light transmission by the appearance of light scattering particles such as large protein aggregates [6].

**Figure 1:**
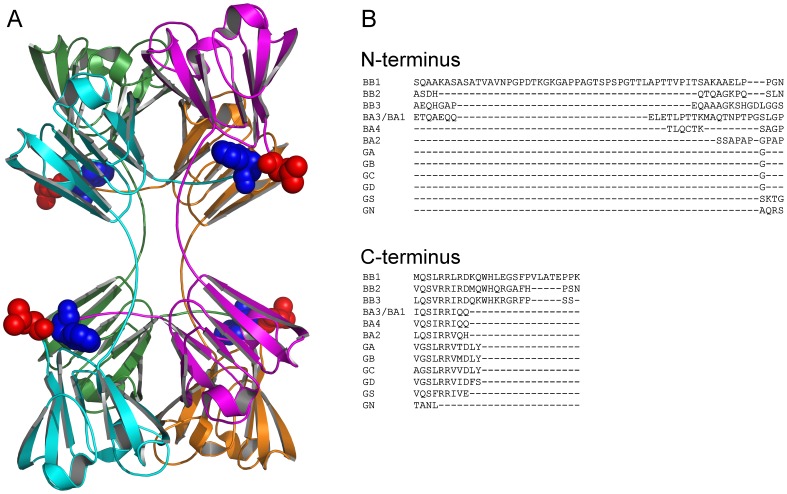
Crystal structure of βB2-crystallin and sequence alignment of β/γ-crystallins. (A) Crystal structure of βB2-crystallin (PDB ID: 2BB2). The four subunits are labeled in cyan, green, orange and magenta, respectively. Leu15 at the N-terminus (red) and Trp195 at C-terminus (blue) are highlighted by the space-filling model to show the role of N-terminus in tetramerization of βB2-crystallin. (B) Sequence alignment of the N- and C-termini of β/γ-crystallins. The sequence alignment was performed using the online software MAFFT (http://www.ebi.ac.uk/Tools/msa/mafft/). The sequences used for alignment are: βB1-crystallin (BB1, P53674), βB2-crystallin (BB2, P43320), βB3-crystallin (BB3, P26998), βA3/A1-crystallin (BA3/BA1, P05813), βA2-crystallin (BA2, P53672), βA4-crystallin (BA4, P53673), γA-crystallin (GA, P11844), γB-crystallin (GB, P07316), γC-crystallin (GC, P07315), γD-crystallin (GD, P07320), γN-crystallin (GN, Q8WXF5) and γS-crystallin (GS, P22914).

β- and γ-Crystallins share a highly conserved tertiary structure, which is composed of four Greek key motifs divided into two domains (Figure 1A). However in the lens, γ-crystallins are monomeric, while β-crystallins exist as various forms of homomers and heteromers ranging from dimer to hexamer or octamer [7], [8]. The highly conserved tertiary structure but quite different quaternary structures of β- and γ-crystallins provides an excellent system to study the evolution and structural determinants of oligomeric proteins. Although the high-resolution structure of β-crystallin heteromer remains elusive, the extensive structural and biophysical studies of β-crystallin homomers and γ-crystallins have revealed that domain swapping and the N- and C-terminal extensions play an important role in β-crystallin oligomerization [7]–[14]. Particularly, compared to γ-crystallins, all β-crystallins have an extra N-terminal extension that range in length from 12 to 57 amino acid residues, and the basic β-crystallins also possess a short C-terminal extensions (Figure 1B) [2]. Among the seven β-crystallins, βB1-crystallin has the longest N-terminal extension, which plays a major role in regulating the size distributions of β-crystallins in vivo by varying the size of N-terminal truncation [7]. Both the NMR and X-ray cryptography studies indicate that the N- and C-termini of β-crystallins are flexible [9]–[11], [15]. However, the N- and C-termini are spatially close to each other in the crystal structures [9]–[11] (Figure 1A), and biophysical studies have indicated that the N-terminal truncation affected the formation of β-crystallin heteromers [12]–[14], [16]. Although it seems clear that the N- and C-termini contributes to β-crystallin oligomerization, structural details remains unclear regarding the key residues involved in protein-protein interactions.

Among the three basic human β-crystallins, βB1- and βB3-crystallins are expressed early during development, while βB2-crystallin is predominant at all stages with a high expression level [17]. Compared to βB1- and βB3-crystallins, βB2-crystallin has a relatively shorter N-terminal extension (Figure 1B). Consistent with the proposal of the role of N-terminus in oligomerization, βB1-crystallin is found to exist preferentially in large heteromers besides the homomeric distributions in human lens [7]. The dissimilar expression patterns and ability to form large oligomers (hexamer and octamer) create a β-crystallin oligomeric size gradient [2]. βB2-Crystallin is distinct among the human β-crystallins because of its high resistance to posttranslational modifications [18], while the other β-crystallins are often extensively modified including various truncations of N-termini [2], [5]. The fact that the N-terminus of βB2-crystallin remains intact during development implies that the N-terminus is extremely important for βB2-crystallin structure and function. Recently, a newly characterized mutation A2V in βB2-crystallin, which is the only one that occurred at the N-terminus of β-crystallins, has been associated with congenital posterior subcapsular cataract in a four-generation Chinese family [19]. In this study, the effect of the A2V mutation on βB2-crystallin structure and stability was investigated by biophysical methods. Our results showed that the mutation did not affect the structure of the dimeric βB2-crystallin, but impaired the ability of βB2-crystallin to form tetramers. Furthermore, we found that the mutation slightly decreased βB2-crystallin stability and promoted aggregation, which might correlate to the late onset of cataract in the patients. The results herein not only help us to understand the molecular mechanism underlying the congenital cataract caused by A2V mutation, but also provide insights into the roles of the N-terminus in βB2-crystallin oligomerization.

## Materials and Methods

### Materials

Dithiothreitol (DTT), ultrapure guanidine chloride (GdnHCl), isopropyl-1-thio-β-D-galactopyranoside (IPTG), sodium dodecylsulfate (SDS), 1-anilinonaphtalene-8-sulfonate (ANS), and bovine serum albumin were obtained from Sigma. Kanamycin was purchased from Amresco. All other reagents were local products of analytical grade.

### Plasmid Constructs and Site-directed Mutagenesis

The total cDNA of human lens was constructed by the standard DNA cloning procedure as described previously [20]. The coding sequence of the wild type (WT) human βB2-crystallin was obtained from the human lens cDNA library by PCR using the following primers: Forward, CCGGATCCATGGCCTCAGATCACCAGAC; Reverse, CGAAGCTTCTAGTTGGAGGGGTGGAA. The PCR product was ligated to the T-simple vector (Takara Corp.) and sequenced. The gene was then inserted in the expression plasmid pET28a. The six-His Tag sequence of pET28a vector was fused to the N-terminus of the open reading frame to facilitate further purification. The mutant was constructed by site-directed mutagenesis using overlap extension polymerase chain reaction (PCR) [21], [22]. The forward and reverse primers were CCGGATCCATGG*T*CTCAGATCACCAGAC and CGAAGCTTCTAGTTGGAGGGGTGGAA, respectively. The PCR-based site-directed mutagenesis was carried out using 10 ng plasmid vector harboring the *CRYBB2* gene, 10 pmol primer, *LA-Taq* DNA polymerase and the buffer supplied with the DNA polymerase. The 25 cycles of amplification was performed as follows: 94°C for 30 s, 60°C for 30 s and 72°C for 30 s. The amplified fragments were inserted into the vector pET28a after digested with BamHI and HindIII, and confirmed by DNA sequencing. The recombinant pET28a plasmid was then transformed into *Escherichia coli* Rosetta (DE3) (Novagen) for expression.

### Protein Expression and Purification

The WT and mutated βB2-crystallins were overexpressed in *E. coli* cells and purified according to the procedures described elsewhere [23]. In brief, the overexpression of the recombinant proteins was induced by 0.1 mM IPTG. After the addition of IPTG, the *E. coli* cells were grown in the Luria–Bertani medium for 4 h at 37°C. Then the cells were harvested and sonicated, and the soluble fractions were separated by centrifugation at 9000 g. The recombinant proteins in the soluble fractions were collected by a Ni-NTA affinity column, and the final products were purified by a Hiload 16/60 Superdex 200 prep-grade column equipped on an ÄKTA purification system. The purity of the final products was over 98% as characterized by SDS-polyacrylamide gel electrophoresis (SDS-PAGE) and size-exclusion chromatography (SEC) analysis. The SDS-PAGE analysis was performed by using 12.5% separating gel in the reducing conditions, while the native-PAGE analysis was carried out using the same conditions of the SDS-PAGE except that the proteins were not denatured by heating and SDS. Unless elsewhere indicated, all protein samples were prepared in buffer A, which contained 20 mM sodium phosphate, 100 mM NaCl, 1 mM DTT, 1 mM EDTA, pH 7.2. The protein concentration was determined according to the Bradford method using bovine serum albumin as a standard [24].

### Spectroscopic Experiments

The circular dichroism (CD) and fluorescence experiments were performed at a given temperature using proteins solved in buffer A. All spectroscopic experiments were repeated at least three times, and the errors were estimated to within 5% as evaluated by the intensities of the spectra. The CD spectra were recorded on a Jasco J-715 spectropolarimeter (Jasco Corp., Tokyo, Japan) with a 1 mm pathlength cell and a resolution of 0.5 nm. The far-UV CD spectra were scanned over a wavelength range of 190–250 nm, while the near-UV CD spectra were in the range of 250–360 nm. The fluorescence spectra were measured on an F-2500 fluorescence spectrophotometer (Hitachi Ltd., Tokyo, Japan) with a 5-nm slit width for both excitation and emission. The intrinsic Trp fluorescence was measured with an excitation wavelength of 295 nm, while the extrinsic ANS fluorescence was examined with an excitation wavelength of 380 nm. For the ANS fluorescence measurements, the samples were prepared by mixing the protein and ANS stock solutions and equilibrated in the dark for 30 min. The final concentration of ANS was 20 µM. The emission spectra were collected over wavelength ranges of 300–400 nm and 400–600 nm for the intrinsic and ANS fluorescence, respectively. The resultant CD and fluorescence spectra were obtained by the subtraction of the spectra of the corresponding buffers. Parameter *A*, which is the characteristic of the shape and position of the fluorescence spectrum [25], was calculated by dividing the intensity at 320 nm to that at 365 nm of the intrinsic Trp fluorescence. The ^1^H-NMR experiments were performed on a Varian Unity Inova 500NB NMR spectrometer at 20°C. The NMR samples were prepared in 10 mM Tris-HCl buffer containing 9% D_2_O and 1 mM DSS. About 500 µl solutions were transferred to a 5-mm diameter NMR tube and equilibrated for 10 min at 20°C before inserting into the NMR probe. The ^1^H-NMR spectra were recorded using a spectral width of 8003.2 Hz (16 ppm) and a recycle delay of 1 s. The NMR data were processed and analyzed using the VNMR software provided by Varian Inc. The protein concentration was 0.2 mg/ml for far-UV CD and fluorescence analysis, 1 mg/ml for near-UV CD experiments and 10 mg/ml for NMR spectroscopy.

### Transmission Electron Microscopy

The transmission electron microscopy (EM) experiments were performed on a Hitachi H-7650B transmission electron microscope. The EM samples were prepared by diluting the 4 M GdnHCl-denatured proteins in buffer A for 10 min, and then the samples were deposited onto a freshly glow-discharged carbon coated copper grid. Negative staining samples were obtained by staining the grid with 1.25% uranyl acetate for 30 s. The pictures were taken using a voltage of 80 kV and a magnification of 70000.

### Size-exclusion Chromatography

The size-exclusion chromatography (SEC) analysis of the protein samples was similar to the procedures described previously [23]. In brief, the gel filtration experiments were carried out on a Superdex 200HR 10/300GL column on an ÄKTA fast protein liquid chromatography. The protein concentrations used for the SEC analysis was ranged from 0.2 to 4 mg/ml. The samples with different protein concentrations were prepared by diluting the stock solutions in buffer A with a given dilution ratio, and then the samples were equilibrated for 2 h at 4°C. The column was pre-equilibrated with buffer A, and then about 100 µl protein solutions were injected into the column. All samples were run at a flow rate of 0.5 ml/min at 16°C. The peak area in the SEC profile was determined by fitting the peaks with one or two Lorentz peak(s) using Origin 8 (OriginLab Corp.) as described previously [26].

### Aggregation Experiments

Details regarding the aggregation experiments were the same as those described previously [23], [27]. Protein aggregation was monitored by measuring the turbidity at 400 nm on an Ultraspec 4300 pro UV/Visible spectrophotometer (Amersham Pharmacia Biotech, Uppsala, Sweden). The thermal aggregation kinetics was measured by heating the samples at a given temperature, and then the turbidity was recorded every 2 s. The temperature was controlled by a water bath. The concentration-dependence of thermal aggregation was studied using a protein concentration range of 0.2–1 mg/ml. The time-course aggregation during refolding was measured immediately after the refolding was initiated by a fast manual dilution (1∶40) of the GdnHCl-denatured proteins into buffer A at 25°C. The final protein concentration was 0.2 mg/ml for aggregation measurements during refolding. The GdnHCl-denatured proteins were prepared by incubating the proteins in buffers containing 4 M GdnHCl at 37°C for 12 h.

### Protein Unfolding by GdnHCl and Heat

Protein folding experiments and data analysis were performed using the same procedure as described previously [28]. The unfolding induced by GdnHCl were performed by incubating the purified proteins in buffer A containing various concentrations of GdnHCl overnight (>16 h) at room temperature. The unfolded samples were then used for spectroscopic analysis. As for thermal unfolding, the protein solutions were heated continuously from 28°C to 86°C. The fluorescence or turbidity data were collected every 2°C after 2 min equilibration at the given temperature. The protein concentration was 0.2 mg/ml for unfolding studies.

## Results

### The A2V Mutation Impairs βB2-crystallin Tetramer Formation, but not the Secondary and Tertiary Structures

**Figure 2:**
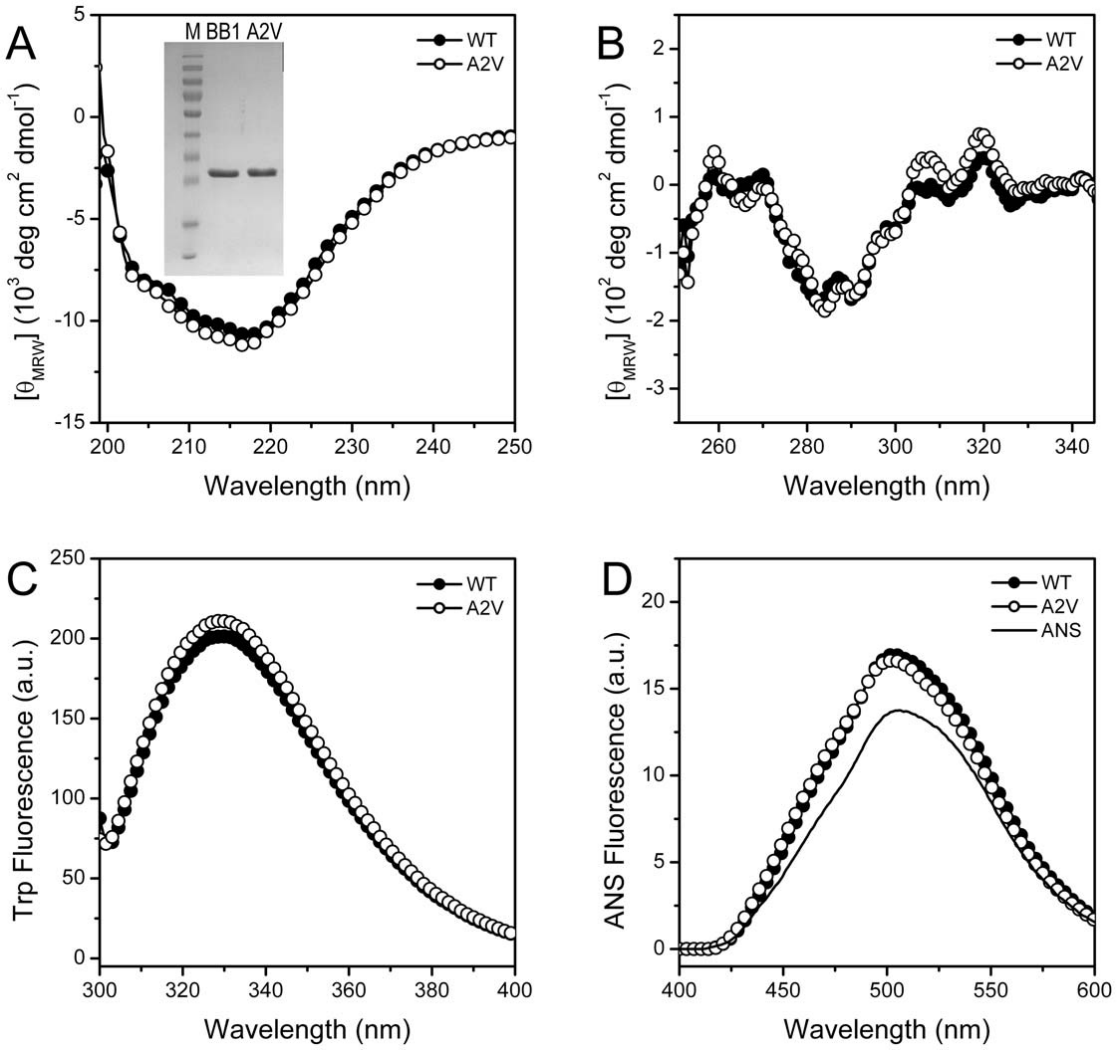
Effect of the A2V mutation on βB2-crystallin structure probed by spectroscopic methods. (A) Far-UV CD spectra. The inset shows SDS-PAGE analysis of the purified recombinant proteins. Lane M is the marker, and the molecular weights of the marker proteins are 170, 130, 95, 72, 55, 43, 34, 26, 17 and 11 kDa, from top to bottom, respectively. The protein concentration for the SDS-PAGE analysis was 1 mg/ml. (B) Near-UV CD spectra. (C) Intrinsic Trp fluorescence with an excitation wavelength of 295 nm. (D) Extrinsic ANS fluorescence with an excitation wavelength of 380 nm. The protein concentration was 0.2 mg/ml. All spectroscopic experiments were performed at 25°C.

Both WT and A2V mutated proteins could be successfully obtained in the soluble fractions of the *E. coli* cells. At low protein concentrations, βB2-crystallin mainly exists as a dimer, and thus CD, intrinsic and extrinsic spectra were determined at 25°C for both proteins to assess the effect of the mutation on βB2-crystallin structure. As shown in Figure 2, the two proteins had nearly identical far-UV CD spectra typically for β-sheet proteins with a negative peak centered at around 217 nm, indicating that the percentages of various secondary structure components were not altered by the mutation. The microenvironments of the aromatic residues in the tertiary structure can be evaluated by near-UV CD or intrinsic Trp fluorescence, and the almost superimposed spectra revealing that the tertiary structure of βB2-crystallin was not influenced by the A2V mutation. Furthermore, the hydrophobic exposure of the proteins was determined by ANS fluorescence. The similar minor increase in the ANS fluorescence intensity at 490 nm indicated that both proteins were well packed with little hydrophobic exposure that could be accessed by the ANS molecule. Thus the spectroscopic experiments at a protein concentration of 0.2 mg/ml indicated that the mutation at the N-terminus did not affect the secondary and tertiary structures of βB2-crystallin.

**Figure 3:**
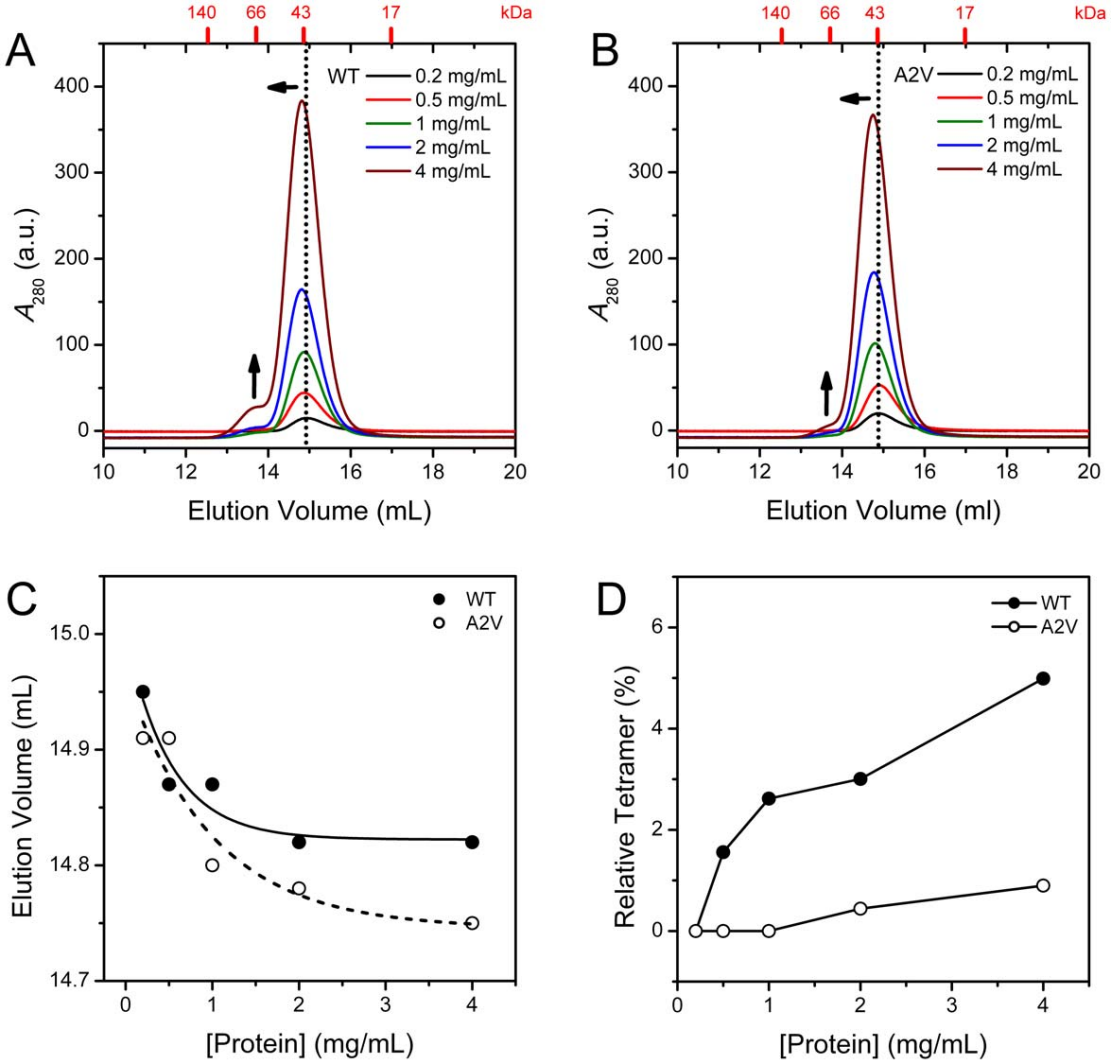
SEC analysis of the WT and mutated βB2-crystallins. (A) SEC profiles of the WT βB2-crystallin. (B) SEC profiles of the A2V mutant. (C) Protein concentration-dependence of the elution volume of the dimer peak. (D) Protein concentration-dependence of the peak area from the tetramers. In panels A and B, the protein concentration-dependent changes of the peaks are indicated by the arrows. The positions of the standard molecular weight markers are shown at the top of the panels A and B. All samples were equilibrated for 2 h at 4°C before SEC analysis.

Previous studies have shown that high-order oligomers can be traced at a high protein concentration of β-crystallins such as in the crystallization conditions [2], [9]. It is worth noting that all β-crystallins are in an oligomeric equilibrium in solutions with the *K*_d_ values at the micromolar level [12]–[14], [16], [29]. The effect of the A2V mutation on βB2-crystallin homo-oligomerization was studied by the concentration-dependence of the SEC profile (Figure 3). At a concentration of 0.2 mg/ml, both of the WT and mutated proteins eluted as a single peak with a molecular weight close to the dimeric form. As protein concentration increased, the main peak shifted to a smaller elution volume accompanied with the appearance of a new peak corresponding to the tetramer. A quantitative evaluation of the oligomeric states was achieved by measuring the concentration-dependence of the peak shift of the dimer peak (Figure 3C) and peak area change of the tetramer peak (Figure 3D). The results clearly indicated that the mutant has a much lower potency to form large oligomers when compared to the WT βB2-crystallin. However, the dimer peak of the mutant showed a relatively larger movement towards the smaller elution volume, which corresponds to a larger apparent molecular weight. This phenomenon suggested that the mutant was more prone to associate into dimers but not tetramers.

**Figure 4:**
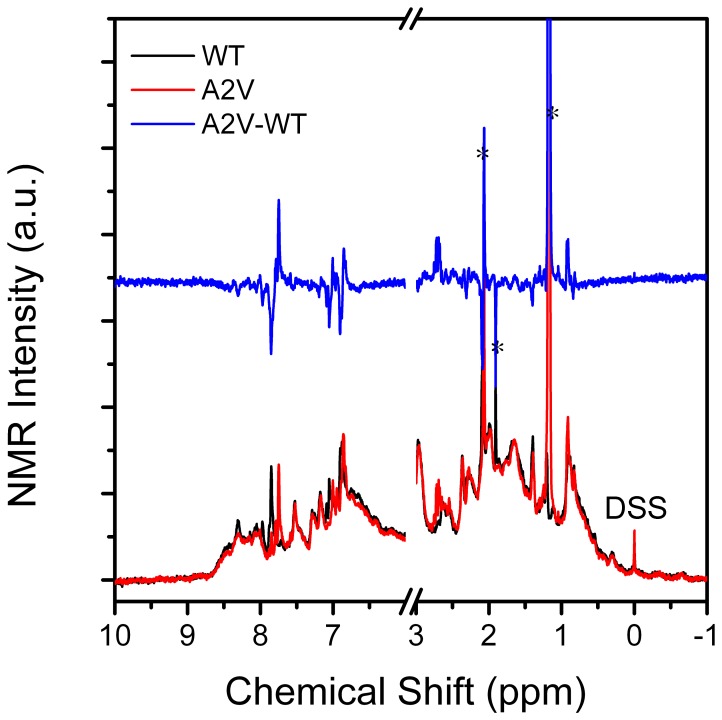
^1^H-NMR spectra of the WT and mutated βB2-crystallins. The 500 MHz ^1^H-NMR spectra were recorded using a protein concentration of 10 mg/ml at 20°C. The chemical shifts were referenced to DSS. The difference spectrum (blue) is obtained by subtracting the NMR spectrum of the mutated protein (red) by that of the WT protein (black). The asterisks indicate the peaks from the buffer.

Since CD and fluorescence spectra are not applicable for high protein concentrations where βB2-crystallin tetramers are stabilized, ^1^H-NMR spectroscopy was recorded at a protein concentration of 10 mg/ml to further confirm the effects of the A2V mutation on βB2-crystallin structure. Although the resolution of the NMR spectra was poor due to the high molecular weights of the oligomers (Figure 4), the similar NMR peak distributions of the two proteins indicated that the overall fold of βB2-crystallin was not altered by the mutation. However, deviations could be observed in the NMR spectra, particularly at the high field region (6–10 ppm), where the peaks are mainly from the backbone NH and aromatic side chains. In the difference spectra, the positive and negative peaks appeared in pairs, suggesting that the positions of some peaks moved to the down field slightly. This implies that the mutation had a minor effect on the structural arrangements of βB2-crystallin. Thus a combination of the CD, fluorescence, SEC and NMR analysis suggested that the mutation did not affect the secondary and tertiary structures, but retard the tetramerization of βB2-crystallin.

**Figure 5:**
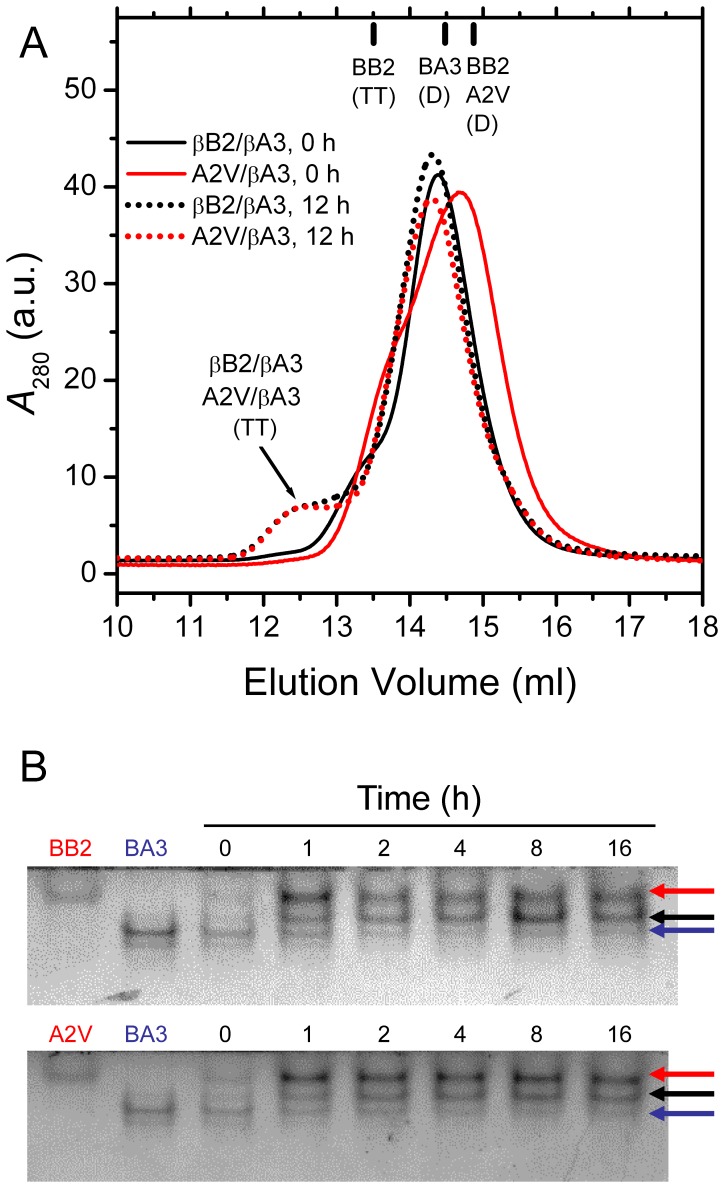
Formation of βB2/βA3-crystallin heteromers probed by SEC and native-PAGE analysis. (**A**) SEC analysis. Equal molar of βB2- and βA3-crystallin solutions were mixed and injected into the column immediately (0 h) or after 12 h equilibration at 37°C. The peak positions of the dimeric and tetrameric homomers are labeled on the top of the plot. D is dimer, and TT is tetramer. (B) Native-PAGE analysis. βB2- and βA3-crystallin solutions were mixed and equilibrated for 0–16 h at 37°C, and then the mixtures were used for native-PAGE analysis. The red, blue and black arrows indicate the bands corresponding to WT and mutated βB2-, βA3- and βB2/βA3-crystallins, respectively.

βB2-Crystallin has the ability to bind with βA3-crystallin to form heteromers [29], [30]. As shown in Figure 5A, the A2V mutation affected the peak position of βB2/βA3-crystallin prepared by fast manual mixing. The peak position of βB2/βA3-crystallin was slightly larger than those of βB2- and βA3-crystallin homodimers, indicating that the WT βB2-crystallin could form heterodimer with βA3-crystallin very quickly. As for the mixture containing the A2V mutant and βA3-crystallin, the main peak eluted at a volume between the homodimers in the SEC profile, while a shoulder appeared at a smaller elution volume. These observations suggested that the WT βB2-crystallin might have a stronger propensity to bind with βA3-crystallin when compared with the A2V mutant. However after 12 h incubation, the SEC profiles of the two proteins were similar with a new peak appeared at about 12.5 ml, which was from the tetrameric heteromer. The similar ability of both WT and mutated βB2-crystallin to bind with βA3-crystallin was also confirmed by the native-PAGE analysis (Figure 5B). The existence of three bands after 16 h equilibration indicated that the proteins were in a dynamic equilibrium between homomers and heteromers, consistent with previous observations [2], [29], [30]. Thus the results in Figure 5 indicated that the A2V mutation did not affect the formation of stable heteromers between βB2- and βA3-crystallins.

### The A2V Mutation does not Affect βB2-crystallin Stability against GdnHCl- and UV-Induced Denaturation

**Figure 6:**
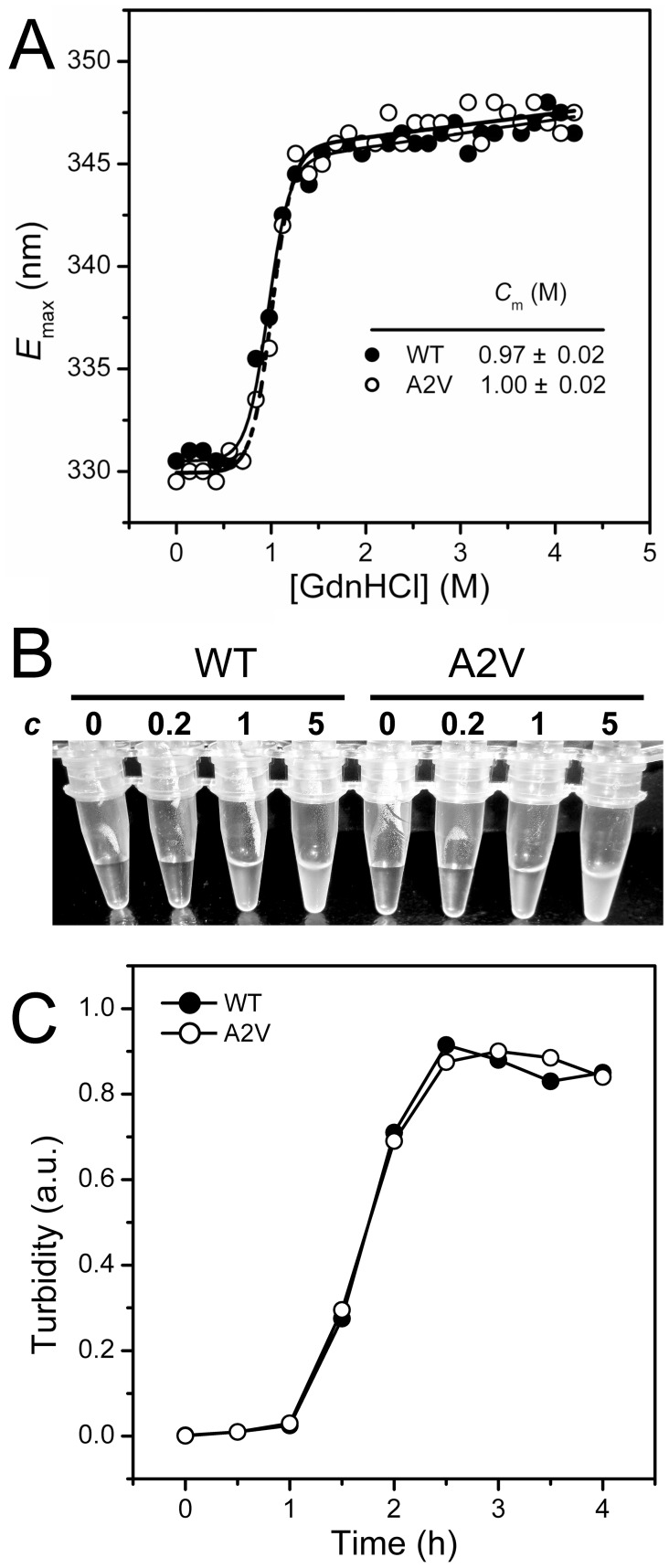
Effects of the A2V mutation on βB2-crystallin structural stability against GdnHCl- or UV-induced denaturation. (A) Unfolding transition curves from the emission maximum wavelength of the intrinsic Trp fluorescence (*E*_max_). The proteins with a protein concentration of 0.2 mg/ml was denatured in buffer A containing various concentrations of GdnHCl overnight. The raw data were fitted by a two-state transition, and the midpoints of unfolding (*C*_m_) are presented. (B) Concentration-dependence of the UV-irradiation induced aggregation. The samples were irradiated by 254 nm UV light for 24 h at 4°C. The protein concentration (*c*) for each sample is labeled above the tube, and 0 denotes the buffer in the absence of proteins. (C) Time-course aggregation induced by UV-irradiation. The protein concentration was 1 mg/ml in buffer A.

Chemical denaturants-induced denaturation is a frequently used method to study the unfolding pathway and structural resistance to stresses in vitro. Although the proteins will not suffer such strong denaturing conditions in vivo, the in vitro studies can mimic the long-term effect of the various stresses and the behavior of the proteins against these stresses. Thus equilibrium unfolding experiments were carried out by using GdnHCl as the denaturants. The unfolding transition curves were well superimposed for both WT and mutated βB2-crystallin when monitored by the maximum emission wavelength of the intrinsic Trp fluorescence (Figure 6A) or ellipticity at 217 nm (data not shown). This observation suggested that the A2V mutation did not affect βB2-crystallin stability against the strong ionic denaturant GdnHCl. Furthermore, UV irradiation is a pathological-correlated stress, and some cataract-linked mutations have been shown to increase the sensitivity to UV damage [27], [28], [31]. As shown in Figures 6B and 6C, neither the WT nor the mutated βB2-crystallin aggregated at a low concentration of 0.2 mg/ml, while similar aggregation behavior was observed for high protein concentrations of 1 mg/ml and 5 mg/ml. This observation suggested that A2V mutation had no impact on the UV irradiation-resistance of βB2-crystallin.

### The A2V Mutation Decreases βB2-crystallin Thermal Stability and Promotes Thermal Aggregation

**Figure 7:**
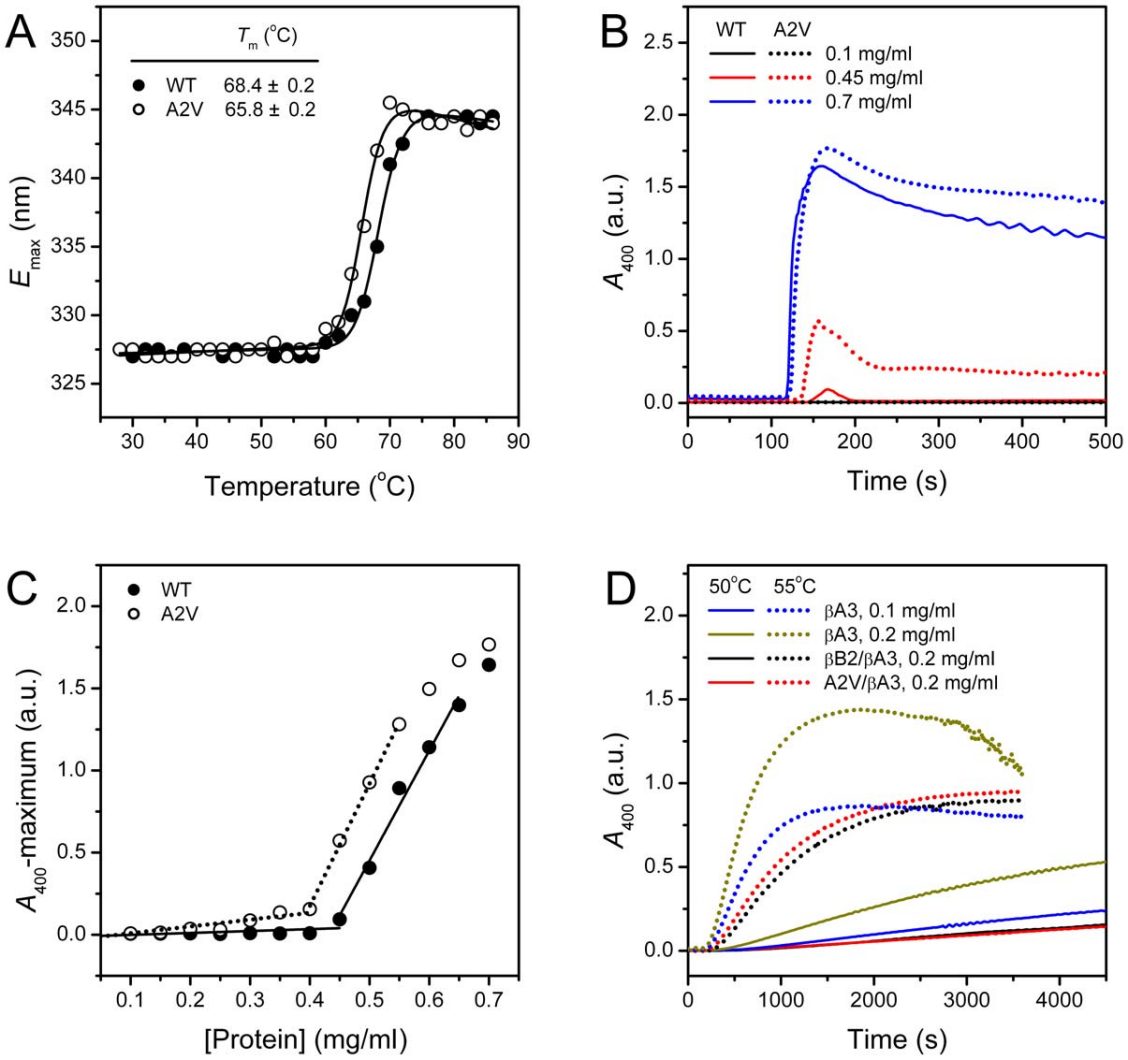
Thermal stability of β-crystallins. (A) Equilibrium thermal transition curves from *E*_max_. The data were fitted by a two-state model, and the midpoints of unfolding (*T*_m_) are shown in the plot. The protein solutions were heated continuously by a water bath from 28°C to 86°C, and fluorescence spectra were recorded every 2°C after 2 min equilibration at the given temperature. (B) Concentration-dependence of the thermal aggregation kinetics. Only the representative kinetic data are presented. The protein solutions were heated at 70°C continuously, and the turbidity data were recorded every 2 s. (C) Relationship between the maximum turbidity and protein concentration. The data were fitted by two linear parts, and the fitting results are shown by lines. The turbidity values above 1.5 were not included in the fitting due to the limitations of the technique. (D) Protection of βA3-crystallin thermal aggregation by βB2-crystallin at 50°C (solid lines) or 55°C (dotted lines). The βB2/βA3-crystallin heteromer was prepared by incubating the mixtures containing equal molar of βB2- and βA3-crystallins for 20 h at 37°C.

**Figure 8:**
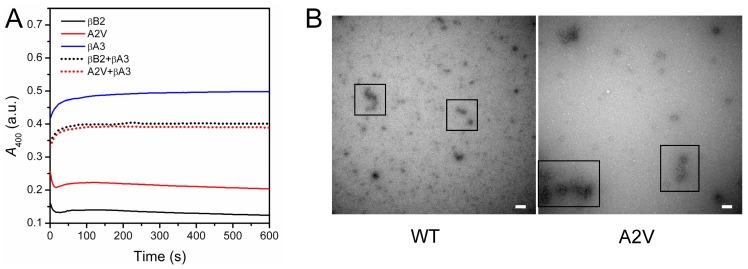
Aggregation of β-crystallins during kinetic refolding. (A) Time-course study of the aggregation of β-crystallins with a final concentration of 0.2 mg/ml. The proteins were denatured by 4 M GdnHCl for 12 h, and refolding is initiated by fast manual dilution (1∶40) of the denatured proteins in buffer A. The dead time of the aggregation experiments was 2 s. (B) Characterization of the morphology of the aggregates formed after 10 min refolding by EM. The bars in the pictures represent 100 nm. The positions of typical aggregates are labeled by open squares.

Protein denaturation by heat treatment usually undergoes dissimilar pathways when compared to denaturation induced by chemical denaturants due to the different nature of stresses. Thus we further studied the effect of A2V on the resistance of βB2-crystallin to heat treatment. As shown in Figure 7A, the thermal transition curves of both proteins followed an apparent two-state process. The mutation decreased the midpoint of the thermal unfolding (*T*_m_) by about 2.5°C. At high temperatures, both proteins aggregated in a concentration-dependent manner (Figure 7B). The thermal aggregation kinetics of both proteins was found to be quite different from the behavior of the other crystallins [27], [32]–[34]. After the lag time, an abrupt increase was observed, and followed by a decrease in the turbidity. EM studies revealed that both proteins formed amorphous aggregates at high concentrations (data not shown, also refer to Figure 8). The cause of this usual aggregation kinetics remains unclear since βA3-crystallin or βB2/βA3-crystallin followed the first-order aggregation kinetics under the same conditions (Figure 7D). A possible explanation is that βB2-crystallin might form large aggregates at the initial stage, and reorganized to smaller or compact ones as the heating time increased. Nonetheless, the data in Figure 7B indicated that the mutant was more prone to aggregate when compared to the WT protein. A quantitative evaluation was achieved by plotting the maximum of turbidity versus protein concentration (Figure 7C). The WT protein did not aggregate at concentrations below 0.4 mg/ml when heated at 70°C. At higher protein concentrations, the maximum turbidity showed an approximate linear relationship to protein concentration. It is worth noting that the deviations from the linear relationship for the *A*_400_ values above 1.5 are caused by the limitations of the absorbance spectroscopy. The mutant showed two linear parts with different slopes in the plot, which was different from the WT protein. Thus it seems that the mutation might interfere with the thermal aggregation mechanism of βB2-crystallin, which further promoted the aggregation of the mutant. βB2-Crystallin has been shown to be able to protect βA3-crystallin against aggregation [35]. Consistent with previous results, we also found that the thermal aggregation of βA3-crystallin was decreased by βB2-crystallin at both 50°C and 55°C. The protective effect of βB2-crystallin on βA3-crystallin thermal aggregation was smaller than that of βB1-crystallin [33], implying that βB1-crystallin had a tighter binding with βA3-crystallin. This deduction is consistent with the fact that βB1-crystallin is more preferentially to exist in the heteromers in the lens [7]. No significant difference was observed for the protective effect between the WT and mutated βB2-crystallin, which is consistent with above result that the mutation did not affect the formation of βB2/βA3-crystallin heteromer.

### The A2V Mutation Promotes βB2-crystallin Aggregation During Kinetic Refolding

To investigate whether the A2V mutation affects the folding of the nascent polypeptides, aggregation during the kinetic refolding was recorded for both proteins. Aggregates appeared immediately for both proteins after the fast manual dilution of the GdnHCl-denatured proteins in refolding buffer (Figure 8). Similar to the thermal aggregation kinetics, the aggregation kinetics during refolding of βB2-crystallin showed differently from normal proteins. Unlike the first-order kinetics of other β-crystallins [28], the turbidity decreased sharply within the first 20 s after the initiation of βB2-crystallin refolding, followed by a slow increase. EM pictures showed that both proteins formed amorphous aggregates with a size range of 100–1000 nm under our conditions, while the size of the mutated proteins was larger than the WT protein. Previously we have shown that βB1-crystallin can completely prevent βA3-crystallin aggregation during co-refolding [28]. However, this protective effect was not observed for βB2-crystallin since the aggregation of a mixture containing both βB2- and βA3-crystallins in equal molar ratio was even larger than the average of the homomers. Meanwhile, the A2V mutant showed similar behavior as the WT protein, regardless of its more serious aggregation when refolded alone. This observation is consistent with above results that the mutation did not affect the formation and stability of βB2/βA3-crystallin heteromers (Figures 5 and 7).

## Discussion

In vertebrate lens, β-crystallins exist as large homomers or heteromers ranging from dimer to hexamer/octamer, which is believed to play an important role in the maintenance of optical properties of lens throughout the individual’s lifespan [2], [7], [8], [36]. Early studies have shown that there existed two (β_H_ and β_L_) or three (β_H_, β_L1_ and β_L2_) distinct assemblies for bovine β-crystallins when analyzed by gel permeation chromatography [37]. The oligomeric states of β-crystallins are regulated by many factors including solution conditions, post-translational modifications and β-crystallin compositions. Among the three human βB-crystallins, *CRYBB1* and *CRYBB3* are early expressed genes, while *CRYBB2* is expressed at a high level at all stages in human lens [2], [17]. The distinct expression pattern of the βB-crystallins is proposed to correlate with the production of a size gradient of βB-crystallins decreased from the nucleus to the cortex [2]. That is, the high expression of βB1-crystallin in the nucleus facilitates the formation of β_H_, while the predominant βB2-crystallin helps the formation of more β_L_ in the cortex. Unlike the dominant heteromeric states of the other β-crystallins, βB2-crystallin is found to exist in both homomers and heteromers in human lens [7]. Another unique property of βB2-crystallin is that it is the most stable one among β-crystallins with high resistance to post-translational modifications [18]. The dissimilar properties of β-crystallins suggest that they play different roles in keeping lens transparency and refractive index.

The highly conserved tertiary structure and distinct quaternary structures of β/γ-crystallins provide a unique model system to study the evolution of oligomerization. The previous extensive studies have shown that the N-terminal extensions of β-crystallins play an important role in the formation of high-order oligomers. Structural studies of βB1- and βB2-crystallins indicated that the N- and C-termini are spatially close to each other [9], [11]. Recent biophysical studies in βB1-crystallin also shown that the N-terminal extension is involved in heteromer formation [13], [14], [16]. However, the structural basis remains unclear for intersubunit interactions of the N- and C-terminal extensions since these extensions are usually cleaved to facilitate crystallization. The recently characterized A2V mutation in βB2-crystallin is the only mutation characterized thus far in the N-terminal extensions of β-crystallins, and thus provides an excellent starting point to study the role of N-terminal extensions in β-crystallin assembly. In this study, we found that the A2V mutation indeed impaired the tetramerization of βB2-crystallin. Since Met1 will be cleaved after translation, Ala2 is the very first residue of βB2-crystallin. The results herein not only support that the N-terminal extension of βB2-crystallin is involved in tetramerization, but also highlight that a minor change in the size of side chains of the first residue could modulate the high-order oligomerization of βB2-crystallin.

The structural basis of the effect of A2V mutation on βB2-crystallin oligomerization remains elusive, while the results herein provide some clues. According to the crystal structure of βB2-crystallin, the N-terminal extension of one subunit is swapped to the C-terminal domain of the other subunit in the dimeric molecule (Figure 1A) [9]. The substitution of the Ala at position 2 by Val will produce two possible effects: the impairment of the interactions required for tetramerization, or the reinforcement of the interactions that stabilize the dimeric structure to shift the tetramer-dimer dynamic equilibrium to dimer. The later one is more likely to be true since the N-terminal extension seems not directly involved in the binding interface of the tetramer formed by two dimers [9]. The inconsistent changes in the elution volume of the dimer peak and the peak area of the tetramer peak in the SEC analysis (Figure 3) suggested that the dimer might be stabilized by the A2V mutation. If the proposal that A2V mutation stabilizes the dimer is correct, it could be further deduced that Ala2 might participate in the dimer formation via hydrophobic interactions of the side chains. The high conservation of Ala2 across species might be the result of a balance between dimer stability and high-order oligomerization during evolution.

The A2V mutation was found to affect βB2-crystallin stabilities against chemical denaturants, UV-irradiation and heat treatment differentially. The mutation did not affect βB2-crystallin stability against GdnHCl- or UV-induced denaturation, but decreased resistance to heat treatment. The dissimilarities in the response to various stresses might be due to the different nature of various chemical or physical denaturation methods. Previous studies have shown that the unfolding of β-crystallins by GdnHCl or urea is a multi-state process involving an intermediate state with the dissociation of the dimer and the unfolding of the N-terminal domain [28], [38]–[40]. The mutant A2V had an oligomeric equilibrium similar to the WT βB2-crystallin at low protein concentrations, and thus it is possible that the mutation did not influence the unfolding pathway and energetics of βB2-crystallin when denatured by chemical denaturants (Figure 6). However, the mutant was more prone to aggregate during kinetic refolding (Figure 8), which might be caused by partial reversibility of βB2-crystallin folding and facilitation of the off-pathway aggregation caused by the increase of hydrophobicity. The behavior of β-crystallin thermal denaturation is an apparent two-state irreversible process with aggregation occurs at high temperatures [33]. When temperature increased from 5°C to 35°C, the dissociation constants of dimeric β-crystallins decreased and the dimer is stabilized [12]. Although no values are available for large β-crystallin assemblies, the formation of high-order oligomers is proposed to be important to protect crystallins from crystallization or deposition in the lens [2]. The decrease in thermal stability by the A2V mutation, which impaired βB2-crystallin tetramerization, strongly suggested that the high-order oligomers might be energetically favored at physiological temperatures. Although the aggregation kinetic parameters were unable to be determined due to the unusual behavior of βB2-crystallin aggregation, it is clear that the mutation could promote βB2-crystallin thermal aggregation as evidenced by the larger values of turbidity.

The decrease in βB2-crystallin thermal stability (2.5°C) was minor when compared to the other well-studied mutations in crystallins. For example, the G61C mutation in γD- crystallin and G129C mutation in γC- crystallin do not significantly affect the structures of proteins, but lead to about 6°C and 16°C decrease in *T*_m_, respectively [27], [34]. Similar to the A2V mutation in βB2-crystallin, the S129R mutation in βB1-crystallin does not affect heteromer formation when analyzed by SEC. However, the S129R mutation significantly impaired the protective effect of βB1-crystallin on βA3-crystallin [33], while the A2V mutation in βB2-crystallin was not. The minor changes by the A2V mutation was consistent with the phenotype in the patients, who noticed their optical defects at the twenties [19]. It is possible that the mutation did not lead to obvious injury in the structure, stability and function of βB2-crystallin as well as the lens for the first 20 years of the patients. As age increased, damages in βB2-crystallin would accumulate by the unavoidable physical and chemical stresses. The slight decrease in thermal stability of the mutated βB2-crystallin finally led to the onset of cataract much earlier than normal people, but later than those highly harmful mutations. Our results herein not only provide insight into the molecular mechanism underlying the hereditary cataract caused by the A2V mutation, but also highlight the important role of the very first residue at the N-terminus in βB2-crystallin oligomerization and stability.
